# Editorial: Advancing musculoskeletal injury management in sports medicine through MRI innovations

**DOI:** 10.3389/fspor.2026.1839040

**Published:** 2026-05-12

**Authors:** Rong Lu, Yong Lu, Tamotsu Kamishima, Takeshi Fukuda

**Affiliations:** 1 Department of Radiology, Huashan Hospital, Fudan University, Shanghai, China; 2 Department of Radiology, Ruijin Hospital, School of Medicine, Shanghai Jiao Tong University, Shanghai, China; 3 Department of Radiology, Hokkaido University, Sapporo, Japan; 4 Department of Radiology, The Jikei University School of Medicine, Tokyo, Japan

**Keywords:** anterior talofibular ligament, cartilage injury, magnetic resonance imaging, muscle trauma, musculoskeletal injury, patellofemoral osteoarthritis, sports medicine

As the public increasingly prioritizes a healthy lifestyle, more people are actively participating in various sports and physical activities. However, this surge in participation brings an unavoidable challenge: our musculoskeletal system is being subjected to significant mechanical stress. From common joint wear-and-tear and ligament sprains to microscopic trauma within the muscles, these sports-related injuries not only affect athletes' performance but also trouble everyday fitness enthusiasts.

To ensure people can safely and sustainably enjoy the benefits of exercise, we must keep their bones and muscles functioning smoothly. This requires more precise tools in sports medicine to detect joint and muscle damage early, accurately track tissue healing, and plan better surgical strategies when conservative treatments fail. Fortunately, the rapid advancement of advanced imaging technologies—such as 3D modeling, machine learning, and functional magnetic resonance imaging (MRI)—has provided researchers with entirely new concepts and tools to monitor, rehabilitate, and protect the body during active living. This special issue brings together five highly practical studies that demonstrate how MRI innovations are transforming the management of sports injuries.

In weight-bearing exercises, the knee is often the first joint to complain. Lyu et al. delved into patellofemoral osteoarthritis (PFOA), a condition prevalent among active individuals. While traditional MRI can clearly show severe, late-stage joint damage, the research team wanted to push the early warning window much further forward. By combining a machine learning-based radiomics approach with Q-Dixon MRI technology, they conducted a detailed analysis of the quadriceps fat pad. Through the analysis of fat fraction data, their predictive model accurately distinguished knees with PFOA from healthy ones. This non-invasive diagnostic approach means doctors can catch degenerative changes early on, allowing patients to adjust their exercise routines before irreversible joint damage occurs.

Moving down the leg, frequent physical activity often leads to ankle sprains. During an inversion ankle sprain, the anterior talofibular ligament (ATFL) is the most vulnerable structure. Although many people recover simply with rest, up to 40% of patients eventually develop chronic ankle instability and require surgical intervention. To help surgeons better understand and manage these injuries, He et al. tested the feasibility of reconstructing a three-dimensional model of the ATFL using 3D MRI. Instead of being limited to flat 2D MRI slices, doctors can use this 3D model to obtain highly precise measurements of the ligament's length, width, and thickness. This provides surgeons with a much clearer, intuitive blueprint for preoperative planning, making ligament repair and reconstruction surgeries far more precise.

Sometimes, however, ankle injuries penetrate deeper into the bone and cartilage, necessitating complex procedures like autologous osteochondral transplantation (AOT). Liu et al. tracked patients who underwent this surgery for severe talar lesions. A major challenge doctors face post-surgery is confirming whether the cartilage is healing properly. The researchers compared standard MRI evaluation scores (MOCART) with the patients' actual physical function and direct visual assessments from second-look arthroscopy. Interestingly, they found that the imaging scores had surprisingly low correlations with how well the patients could walk or what the inside of the joint truly looked like. This offers a crucial takeaway for the medical community: while MRI is an exceptional tool, it requires more refined evaluation criteria and absolutely cannot fully replace direct clinical evaluation when assessing cartilage repair.

Beyond the joints, muscle health is equally vital. Running marathons is a popular way to stay fit, but long-distance running causes microscopic tears in the leg muscles. In a comprehensive mini-review, Cheng and Li detailed how advanced quantitative MRI technologies can track this lower extremity muscle microtrauma. They pointed out that techniques like T2 mapping can pinpoint inflammatory edema, while Diffusion Tensor Imaging (DTI) can detect subclinical muscle fiber tears. They also highlighted the use of Magnetic Resonance Spectroscopy (MRS) in tracking the consumption of lipid fuels within the muscles. By monitoring these microscopic changes, sports medicine experts can use objective data to advise runners on when it is truly safe to return to the track, thereby preventing minor soreness from escalating into a severe muscle tear.

Finally, upper body sports injuries cannot be ignored. Teng et al. investigated shoulder pain caused by rotator cuff muscle degeneration. When patients undergo physical rehabilitation, it is usually difficult to objectively measure whether the muscle is healing internally. Using IVIM and FACT MRI techniques, they evaluated patients before and after three months of rehab exercises. The study found that impaired capillary blood flow—measured by D* values—is a much more sensitive indicator of early muscle degeneration than fat infiltration. Even more encouraging, after the rehabilitation period, these D* values in the patients' supraspinatus muscles significantly increased, which directly correlated with their reduced pain and improved shoulder function. This not only proves that targeted sports rehabilitation can physically restore microcirculation in damaged tissues but also offers a new, non-invasive way to monitor rehabilitation outcomes.

Ultimately, we cannot simply encourage people to “exercise more” without providing a solid medical safety net for their bones, joints, and muscles. The studies in this collection compellingly demonstrate that advanced imaging technology is the core of this safety net. Whether it is using machine learning to catch early knee damage, rendering 3D models to repair an unstable ankle, or tracking blood flow changes in a healing shoulder, these cutting-edge MRI technologies have built a bridge that allows people to stay active while staying away from harm. By moving from simple visual scans to highly detailed quantitative diagnostics, we can better guide sports rehabilitation and surgical interventions. This not only advances the field of sports medicine but also ensures that every sports enthusiast receives comprehensive protection and support for their body.

